# Microalgal starch: regulation of its quantity and roles

**DOI:** 10.3389/fpls.2026.1736651

**Published:** 2026-01-30

**Authors:** Imran Pancha, Sousuke Imamura

**Affiliations:** 1 Department of Industrial Biotechnology, Gujarat Biotechnology University, Gandhinagar, Gujarat, India; 2 Space Environment and Energy Laboratories, NTT, Inc., Tokyo, Japan

**Keywords:** carbon partitioning, environmental factors, metabolism, microalgae, starch, target of rapamycin

## Abstract

Algae are important organisms that fix atmospheric carbon dioxide and produce organic compounds via photosynthesis. Among these, starch serves as the principal storage polysaccharide in algae and plays a crucial role in the production of various industrially relevant bioproducts, such as biofuels, animal feed, and bioplastics. This review article summarizes the functions of starch in microalgae and the impact of environmental factors, such as light and nutrient supply, on starch accumulation. Furthermore, it explores how microalgae regulate starch accumulation under specific conditions and introduces recent studies on the signaling pathways and related protein functions involved. Based on these insights, this review article discusses the future research challenges that need to be addressed. Such fundamental research findings form the foundation for applied research aimed at sustainable resource utilization, and are expected to provide crucial insights that will drive innovation in next-generation biotechnology.

## 1 Introduction

Microalgae are promising bioresources for addressing various environmental issues, including global warming, owing to their exceptionally high photosynthetic capacity, rapid growth rate, and ability to grow in diverse environments, especially when compared to terrestrial crop plants. Additionally, microalgae can grow in seawater and obtain nutrients from various types of wastewater (Chisti, 2007). Considering these benefits, various research groups worldwide have attempted to produce green and sustainable raw materials using microalgae for industrial applications (Rizwan et al., 2018; Yu et al., 2024). Furthermore, the relatively simple cellular structure of microalgae combined with recent advances in genetic engineering facilitate the manipulation of their metabolic pathways, thereby enhancing the production of industrially valuable metabolites (Banerjee et al., 2016; Ng et al., 2020). These features make microalgae ideal platform organisms for a wide range of biotechnological applications, such as the production of biofuel feedstocks, high-value nutritional supplements, pharmaceutical ingredients, and precursors for bioplastics (Khan et al., 2018).

Microalgal biomass is primarily composed of lipids, carbohydrates, proteins, and pigments (Arenas Colarte et al., 2025), all of which have potential industrial applications. Among lipids, triacylglycerols (TAGs) are energy storage molecules that have garnered significant attention as a feedstock for biodiesel production, as they offer a renewable alternative to fossil fuels. TAGs typically accumulate under nutrient-depleted conditions (see below), whereas in nutrient-replete conditions, structural lipids such as glycolipids and phospholipids are predominant. Carbohydrates such as starch can be fermented to produce bioethanol or bioplastics (Six et al., 2024; Brouchon et al., 2025), thereby reducing the reliance on petroleum-based polymers (Yamaguchi et al., 2017). It has to be noted here that microalgal cells do not contain lignin in their cellular components. Lignin, which is found in higher plants and seaweeds, poses a challenge to the use of these bioresources in biofuel production (da Maia et al., 2020). The complex nature of lignin complicates the pretreatment processes, and during hydrolysis, it generates inhibitors that can hinder the growth of fermentative microorganisms. In microalgae, the cell walls can be disintegrated by mechanical or chemical process to open up the cells. After that, a simple acid hydrolysis or treatment with enzymes such as amylase is sufficient for the hydrolysis of cellular starch (Delrue et al., 1992; Markou et al., 2012; Six et al., 2024). Microalgae also contain accessory pigments such as carotenoids and phycobiliproteins. Because of their antioxidant, anti-inflammatory, and coloring properties, these pigments have broad applications in the food, cosmetic, and pharmaceutical industries (Sonani et al., 2016). Microalgal proteins are increasingly recognized as sustainable sources of nutrition, with potential applications in animal feed, aquaculture, and human dietary supplements (Janssen et al., 2022).

**Figure 1:**
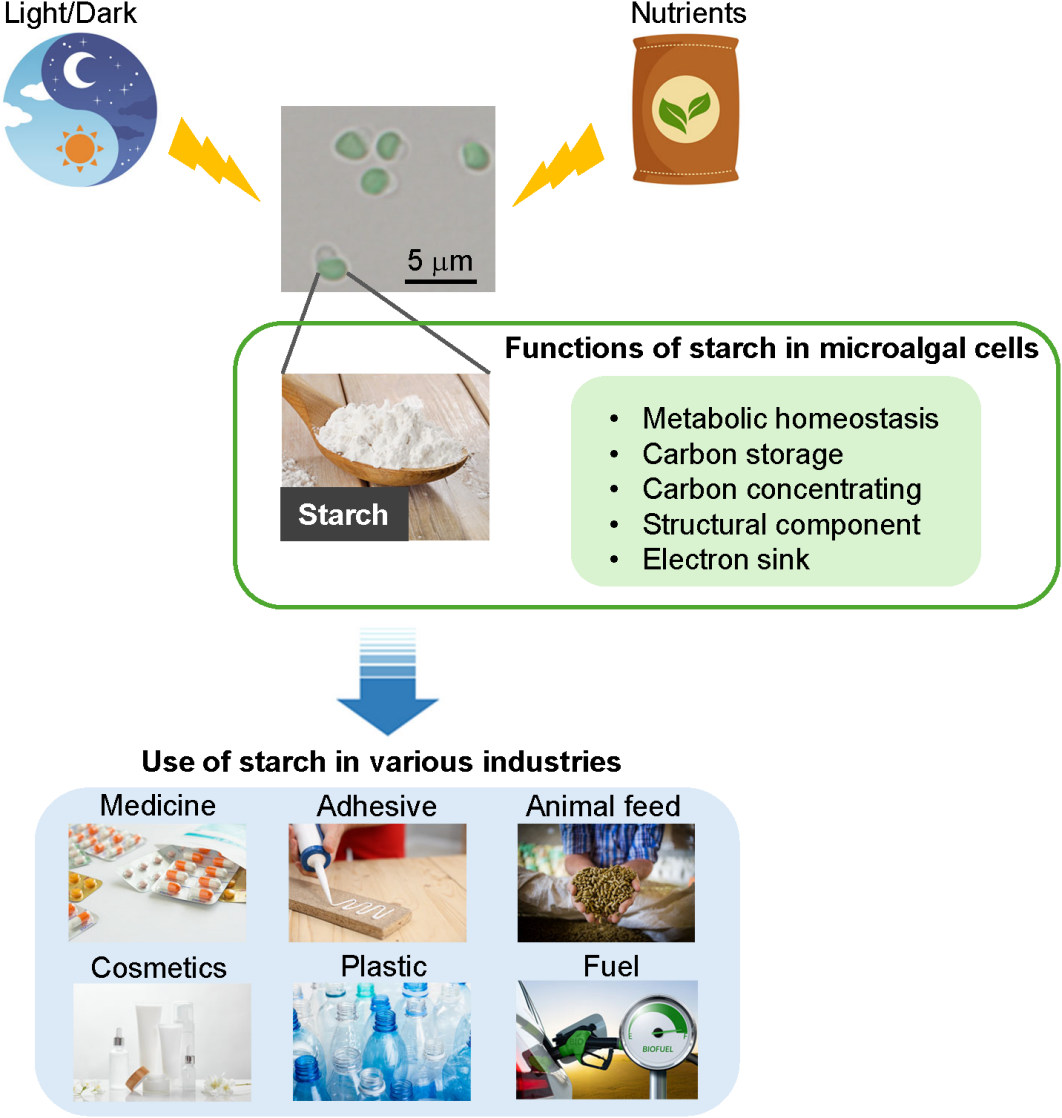
Summary of the functions and applications of starch. Starch accumulation in microalgae in response to light and nutrients. They have several functions and can be used in various industrial applications.

This review focuses on various strategies to improve starch accumulation and its regulation in eukaryotic microalgae and discusses both past and recent findings. However, why is starch important? Beyond the reasons mentioned earlier, starch is a vital component closely linked to our daily lives. For example, starch serves as a raw material for the industrial production of fuels, animal feed, plastics, cosmetics, adhesives, and pharmaceuticals (**Figure 1**). Starches are mainly obtained from crops such as maize and soybeans. However, the issue of food scarcity remains unresolved and is becoming increasingly severe each year. Starchy foods are a major source of carbohydrates in the diet and contribute significantly to caloric intake. Estimates suggest that this contribution accounts for approximately 80% of total global caloric intake (Rashwan et al., 2024). Therefore, it is necessary to reassess the industrial use of crop-derived starch, which is a crucial source of calories. In contrast, microalgae-derived starch can be considered a good alternative to plant-based starch because microalgae accumulate high amounts of starch in their dry cell weight. In addition, as previously mentioned, microalgae do not compete in land-based cultivation. This review of starch accumulation and its regulation in microalgae aims to provide foundational information for addressing these challenges and environmental issues.

## 2 Role of starch in microalgal cell

Starch is a polymer composed of linked glucose monomers. In microalgae, starch can be classified into several types (Busi et al., 2014). Green microalgae, such as *Chlamydomonas*, accumulate starch composed of amylose and amylopectin, which are structurally similar to the starch accumulated by land plants (Ball, 2002). In contrast, red microalgae, such as *Cyanidioschyzon merolae*, accumulate floridean starch, which is mainly composed of biopolymers of amylopectin, which is similar to glycogen (Busi et al., 2014). Furthermore, the intracellular organelles in which starch accumulates in microalgae vary depending on the type. In red microalgae, starch accumulates in the cytoplasm, whereas in other algae, it accumulates in chloroplasts (Hirabaru et al., 2010). Apart from green and red microalgae, diatoms store carbohydrates mainly in the form of chrysolaminarin, a soluble β-(1, 3)-(1, 6)-glucan polysaccharide. In diatoms, chrysolaminarin is synthetized in the cytoplasm and stored in vacuoles (Caballero et al., 2016; Huang et al., 2018; Kwasiborski et al., 2025). In the present manuscript, we mainly focus on the regulation of starch metabolism by green and red microalgae.

Starch is a major source of chemical energy in microalgae and other photosynthetic eukaryotes, and plays a critical role in their metabolic and physiological processes and overall survival (da Maia et al., 2020) (**Figure 1**). Starch is a readily available energy reserve that can be mobilized to meet the cellular demands for growth, maintenance, and stress responses. The synthesis and degradation of starch are tightly regulated in response to environmental cues and metabolic signals, ensuring that energy is efficiently stored and utilized (see the next section) (Geigenberger, 2011). In microalgae, starch metabolism is closely linked to photosynthesis, with excess glucose being converted into starch and stored during the daytime. This conversion helps microalgal cells maintain their cellular osmotic balance and metabolic regulation (Gustavs et al., 2010; Geigenberger, 2011; Decamp et al., 2023). The stored starch can then be broken down to provide energy during periods of darkness or nutrient starvation, allowing cells to sustain their metabolic activities. Apart from metabolism and cell survival, microalgal carbohydrates are important structural components that play a major role in the cell walls of microalgae (Spain and Funk, 2022).

In most of the literature starch is mainly discussed as semi-crystalline granules; however, starch can also be present in different forms. Carbon-concentrating mechanisms (CCMs) are important for the growth and carbon fixation by cyanobacteria and microalgae. In microalgae, the use of carbon-concentrating mechanisms (CCMs) has evolved to concentrate CO_2_ because of its lower solubility and consequently reduced availability (An et al., 2023). Pyrenoids are unique protein complexes found in the chloroplasts of eukaryotic algae that generally contain important enzymes for CO_2_ fixation, i.e., Rubisco (Kuchitsu et al., 1991). In microalgae, starch that accumulates in chloroplasts is directed towards pyrenoids. The starch sheath surrounding pyrenoids plays a critical role in facilitating CO_2_ diffusion within algal cells. Itakura et al. (2019) demonstrated that Starch Granules Abnormal1 (SAGA1), a protein associated with pyrenoids, directly interacts with Rubisco. In the *saga1* mutant, pyrenoids exhibit multiple abnormalities, including the absence of a tubule network and the presence of irregular starch plates. Under low CO_2_ conditions, these irregular starch plates become more pronounced. Collectively, these findings indicate that the morphology of the starch sheath strongly influences both pyrenoid organization and the efficiency of the CO_2_-concentrating mechanism (CCM). In most photosynthetic organisms, ATP generation, without the net production of NADPH, occurs through cyclic electron flow (Alric, 2010). In *Chlamydomonas*, proton gradient regulation 5 (PGR5) is one of the major players. *Chlamydomonas* carrying a mutant *pgr5* has low chloroplast ATP/NADPH ratio. Additionally, this strain does not accumulate starch under nitrogen starvation conditions. This indicates that the *pgr5* mutant re-allocates starch in the cells for cellular maintenance (Saint-Sorny et al., 2024). Under challenging environmental conditions, microalgae such as *Chlamydomonas* develop various mechanisms to manage electrons in various alternative pathways. Saroussi et al. (2019) reported that in *Chlamydomonas*, when cells are transferred from dark to light, electron flow assists in the synthesis of ATP and NADPH within the cells. However, in the starch synthesis mutant (*sta6*), the majority of electrons are directed towards the synthesis of H_2_O via the actions of flavodiiron proteins (FLVs) and plastid terminal oxidases (PTOX). This study indicates that the starch synthesis lies at the intersection of carbon and energy management in the microalgae.

## 3 Conditions under which starch accumulates in microalgae

### 3.1 Changes in day and night

In most photosynthetic organisms, carbon is directed to storage compounds (e.g., carbohydrates and lipids) during the light period and then consumed during the dark period to support various metabolic processes at night (Geigenberger, 2011). Similarly, in microalgae, as in terrestrial plants, starch accumulation generally increases during the day and decreases at night. When the green alga *Acutodesmus obliquus* is grown under a light-dark (LD) cycle, starch metabolism is closely synchronized with the LD cycle (León-Saiki et al., 2017). Starch accumulates during the latter half of the light period and is consumed throughout the dark period. Starch accumulation begins 7 h after ‘sunrise’, simultaneously with a decrease in biomass productivity, reaching a maximum content of 0.20 g/g dry weight by the end of the light period.

In the red microalga *Cyanidioschyzon merolae* (Matsuzaki et al., 2004), after transitioning to darkness under continuous light conditions, the starch content changed 0.66-fold and 0.56-fold compared to the initial levels at 0 h after 6 and 18 h of darkness, respectively (Komiya et al., 2025). Subsequently, 4 h after light re-illumination, the starch content was nearly the same as that at 0 h. Although starch content in algae changes under light/dark (L/D) conditions, the specific mechanisms governing its increase and decrease (synthesis and degradation) during L/D cycles remain largely unclear, and many missing links still need to be uncovered.

### 3.2 Nutritional deficiencies

Changes in starch accumulation under nutrient-deficient conditions have been observed in the green alga, *Chlamydomonas reinhardtii* (Ball et al., 1990). The study indicates cells accumulate high amount of starch after one week of phosphate, nitrogen, or sulfur deprivation compared to nutrient replete conditions. This accumulation occurs irrespective of phototrophic, mixotrophic, or heterotrophic growth conditions. Potassium or magnesium deficiency led to starch accumulation, whereas calcium deficiency did not. Experiments using inhibitors such as streptomycin, which inhibits organelle translation, and cycloheximide, which inhibits cytoplasmic protein synthesis, have indicated that starch accumulation depends on post-transcriptional regulation. Notably, cells that reach the stationary phase, which can be caused by factors such as nutrient depletion or high cell density, even after growth in nutrient-replete media, do not accumulate starch (Ball et al., 1990). This phenomenon is consistent with the fact that in the red microalga *C. merolae*, the amount of starch accumulation remains almost unchanged during culture growth (Takusagawa et al., 2016), as described in the following paragraph. However, in the study conducted by Eriksen et al. (2007), the temporal relationship between nitrogen sources (ammonium) and starch content was analyzed (Eriksen et al., 2007). The starch content increased sharply at almost the same time as ammonium depletion. This supports the idea that nitrogen depletion induces the activation of a signal for starch accumulation, leading to starch accumulation.

In the red microalga *C. merolae*, starch accumulation occurs under nitrogen-deficient conditions (Takusagawa et al., 2016). Although starch levels did not change under nitrogen-sufficient conditions, they increased rapidly between 12 and 72 h under nitrogen-deficient conditions. The maximum level reached was approximately ten times that at the 0-hour point, and this level was maintained until the end of the cultivation period (120 h after nitrogen depletion). Under the conditions of maximum accumulation (96 h after nitrogen depletion), approximately 40 µg of starch accumulated per 10^8^ cells. In that study, authors investigated the expression of genes involved in starch synthesis and degradation. The genes analyzed included UTP-glucose-1-phosphate uridyltransferase, glycogenin, and 1, 4-alpha-glucan branching enzymes for starch synthesis and glycogen phosphorylase, isoamylase, and beta-amylase for starch degradation. However, no significant changes were observed in the expression of these genes in the presence of starch. Additionally, microarray data under nitrogen-deficient conditions showed no change in the expression of genes related to carbohydrate metabolism (Imamura et al., 2015, 2016; Pancha et al., 2019a). These findings indicate that similar to that in *C. reinhardtii*, the regulation of starch levels in the red microalga *C. merolae* is not controlled at the transcriptional level.

In the case of the industrially relevant microalga *Chlorella*, Brányiková et al. (2011) reported that it accumulates substantial amounts of starch under various cultivation conditions. Under high light intensity (330 μmol m^-^² s^-^¹), cells accumulated almost 40% starch of dry cell weight (DCW). They also demonstrated that the cell cycle phase plays an important role in starch synthesis in this microalga. *Chlorella* synthesizes up to 45% starch of DCW before the onset of cell division. When the cytoplasmic protein synthesis inhibitor cycloheximide, which also prevents nuclear division, was added to the cultivation medium, cells accumulated nearly 60% starch. A similar level of starch accumulation was observed under sulfur limitation (Brányiková et al., 2011).

Sulfur (S) is an essential macronutrient required for the synthesis of cysteine, methionine, coenzymes, iron–sulfur proteins, and diverse metabolites in microalgae. During limited sulfur availability, microalgae undergo physiological and metabolic adjustments to maintain viability. A well-documented response to sulfur deprivation is enhanced accumulation of storage carbohydrates, particularly starch, making sulfur stress a practical strategy to boost carbon storage and feedstock quality for biofuels (Melis et al., 2000; Siaut et al., 2011). Under normal growth, photosynthetically fixed carbon is directed into protein synthesis, growth, and maintenance. Sulfur starvation rapidly reduces protein synthesis because sulfur-containing amino acids cannot be produced (Yamazaki et al., 2018), resulting in an excess of fixed carbon that can no longer be used for growth and is consequently rerouted into starch as a protective, energy-dense reservoir (Yamazaki et al., 2018). In *C. reinhardtii*, sulfur starvation triggers increases in starch granule size and abundance within chloroplasts, while chlorophyll content and photosynthetic capacity decline, collectively shifting metabolism from growth to survival (Melis et al., 2000).

The signaling and regulatory pathways governing starch accumulation during sulfur stress involve alterations in carbon-partitioning enzymes. S-starved cells upregulate enzymes associated with starch biosynthesis, such as ADP-glucose pyrophosphorylase (AGPase), while downregulating those involved in starch degradation (Siaut et al., 2011). In parallel, sulfur deprivation impairs synthesis of sulfur-rich components of the photosynthetic apparatus—particularly the D1 protein of Photosystem II—progressively reducing photosynthetic electron flow and thus ATP/NADPH generation. With fewer reducing equivalents available for lipid biosynthesis, carbon is preferentially allocated to starch formation rather than fatty acid synthesis, reinforcing carbohydrate storage during stress.

A two-stage cultivation strategy has emerged as an effective bioprocess to enhance both biomass production and storage metabolite accumulation (e.g., starch). In this approach, cultures undergo an initial growth phase under nutrient-replete, moderate light/CO_2_ conditions to maximize biomass, followed by a stress phase (nutrient limitation and intensified light/CO_2_) to trigger storage compound accumulation. For *Chlorella* sp. AE10, cultures initially grown under moderate CO_2_ (1%), moderate light, and sufficient nitrate for three days were shifted to high CO_2_ (10%), very high light, and nitrogen starvation; by day 5 of the second stage, carbohydrates reached ~77.6% DCW, with starch specifically at ~60.3% DCW, and starch productivity reached ~0.311 g L^-^¹ day^-^¹ (Cheng et al., 2017). The underlying rationale is that, after achieving high cell density, nutrient limitation especially nitrogen starvation suppresses growth-related metabolism (e.g., protein synthesis) while photosynthesis continues to fix carbon; the resulting excess fixed carbon is redirected into starch synthesis. In AE10, the combined stressors (nitrogen limitation + high CO_2_ + high light) efficiently partitioned carbon toward starch rather than growth (Cheng et al., 2017). Apart from this, it should also be noted that photon availability per cell is a key driver of starch accumulation during nutrient starvation. Additionally, under nutrient starvation, reducing the cell density might be also responsible for enhancing volumetric starch productivity (Six et al., 2025).

Similar two-stage or nutrient-shift strategies have been reported in other species. For example, in *C. zofingiensis* under nitrogen starvation, rapid carbohydrate and starch accumulation occurred; over a five-day culture, starch productivity achieved values suitable for bioethanol feedstock considerations (Zhu et al., 2014). These process designs highlight the translational potential of controlled nutrient stress to engineer carbon allocation toward fermentable carbohydrates at industrially relevant scales. However, the two-stage approach requires careful optimization. The nature of the stress and the abruptness of condition changes must be controlled; severe stress may cause cell death, loss of viability, or inefficient conversion of biomass to storage compounds. Additionally, depending on species, carbon partitioning may shift toward lipids rather than starch during prolonged nutrient deprivation. For instance, in *C. sorokiniana* under nitrogen depletion, starch accumulated initially but was partly degraded over time as lipids increased, indicating a temporal shift in storage metabolism (Li et al., 2015).

## 4 Mechanisms regulating starch accumulation in microalgae

### 4.1 Target of rapamycin-signaling regulates starch levels

Starch accumulation is thought to be induced when changes in light conditions or nutrient deficiencies act as cues. The discovery of the mechanisms linking these cues to starch accumulation in photosynthetic eukaryotes was first elucidated in the terrestrial plant A. thaliana, as demonstrated by Moreau et al. (2012) and Caldana et al. (2013).

Moreau et al. (2012) investigated the function of the TOR pathway, a major regulatory factor that senses information about the external environment and intracellular conditions to regulate growth in all eukaryotes (Laplante and Sabatini, 2012). TOR, named for “Target of Rapamycin, “ was originally discovered as the cellular target of the macrolide compound rapamycin, which inhibits TOR signaling (Heitman et al., 1991). However, land plants, including Arabidopsis, are generally insensitive to rapamycin (Menand et al., 2002). It has been verified that TOR forms a complex with proteins such as LST8, which is essential for TOR activity in yeast and animals (Wullschleger et al., 2006). Consistent with these findings, studies in Arabidopsis have demonstrated that TOR also forms a functional complex with LST8, and loss of LST8 severely impairs TOR activity, leading to defects in growth and metabolic regulation, indicating that LST8 is essential for TOR signaling in plants (Moreau et al., 2012; Caldana et al., 2013). To further dissect TOR function, they used mutants of LST8, a component of the TOR complex, to analyze its role in plant growth, flowering, and metabolic adaptation to long-day conditions. In their analysis, it was reported that starch levels were similar between the wild type and the *lst8* mutant line at the end of short-day (SD) conditions (8 h of light) but significantly increased under long-day (LD) conditions (16 h of light) (Moreau et al., 2012). Caldana et al. (2013) demonstrated that the TOR protein not only regulates growth, but also influences nutrient distribution and central energy metabolism using large-scale MS-based metabolite profiling of primary, secondary, and lipid compounds in combination with transcript profiling in lines with conditionally reduced AtTOR expression. They observed a strong increase in starch content across all the examined lines when AtTOR expression was reduced, similar to that observed in the *lst8* knockout mutant (Caldana et al., 2013).

Subsequent studies have investigated the relationship between TOR and starch accumulation in microalgae. Jüppner et al. (2018) explored the role of TOR in biomass accumulation and cell cycle progression in *C. reinhardtii*, which is sensitive to rapamycin in its wild-type form. Their analysis demonstrated that inhibiting TOR in cells growing under light conditions by treatment with rapamycin led to a two-fold increase in starch accumulation compared to control conditions. Conversely, in the dark, 20% of the starch was already degraded within the first hour under control conditions, whereas only 10% was degraded in the cultures treated with rapamycin within the same time frame. This suggests that TOR negatively influences starch accumulation (synthesis) and positively influences starch degradation (Jüppner et al., 2018). Similar results have been observed in the unicellular red microalga *C. merolae* (Komiya et al., 2025) indicating the potential for a common regulatory mechanism in microalgae.

The relationship between TOR and starch accumulation has also been analyzed in the unicellular red microalga *C. merolae*. Since wild-type *C. merolae* does not exhibit sensitivity to rapamycin, the *C. merolae* SF12 strain, which is rapamycin-sensitive, was constructed by expressing yeast FKBP12 within the cells and used in the analysis (Imamura et al., 2013). In this analysis, cells exposed to rapamycin accumulated significantly more starch than the control cells 12 h after exposure. Starch content continued to increase over time, reaching approximately ten times that of the control after 48 h. Moreover, when analyzing starch content under rapamycin treatment, nitrogen deficiency, and a combination of nitrogen deficiency and rapamycin treatment, no significant differences in starch content were observed among the three conditions (Pancha et al., 2019a). This indicates that TOR is the main pathway governing starch accumulation, at least under nitrogen-deficient conditions. These results clearly indicate that TOR signaling is a critical pathway affecting starch content in *C. merolae*, similar to that in *C. reinhardtii*.

#### 4.1.1 Regulation of de novo starch synthesis by TOR-signaling

The mechanism by which TOR regulates starch accumulation determined by the balance between starch synthesis and degradation—has been elucidated in *C. merolae*. This breakthrough was achieved through phosphoproteomic analysis, as transcriptomic data revealed no significant changes in the expression of starch metabolism-related genes under conditions that promote starch accumulation, such as nitrogen depletion and TOR inactivation, not only in *C. merolae* but also in other microalgae (Pancha et al., 2019a; Werth et al., 2019).

Phosphoproteomic analysis using an LC-MS/MS system identified 52 proteins whose phosphorylation status changed following rapamycin treatment. Among these, seven were categorized as carbohydrate metabolism-related proteins, annotated as follows: alpha, alpha-trehalose-phosphate synthase (UDP-forming), fructose-1, 6-bisphosphate aldolase, phosphoglycerate dehydrogenase, probable lipopolysaccharide (LPS) glycosyltransferase, probable phosphoribosyl diphosphate synthetase, ribose-phosphate pyrophosphokinase, probable starch/glycogen synthase, and a protein similar to glycogenin.

Among these seven proteins, two protein similar to glycogenin (hereafter referred to as CmGLG1) and probable starch/glycogen synthase (hereafter referred to as CmGS) were selected for detailed functional analysis because of their similarity to glycogenin and starch/glycogen synthase, respectively, as their homologues play fundamental roles in starch/glycogen synthesis in eukaryotes. Glycogenin initiates starch/glycogen synthesis through autoglycosylation (Roach and Skurat, 1997), a process in which glucose molecules are transferred to glycogenin, forming α-1, 4-glycosidic bonds that create a short primer sequence of approximately 10 to 20 glucose residues (Pitcher et al., 1988). Subsequently, starch/glycogen synthase elongates this primer by adding glucose units to the growing chain (Patron and Keeling, 2005). Functional characterization revealed that overexpression of CmGLG1 resulted in an approximately 4.7-fold increase in cellular starch content compared to the control strain, whereas CmGS overexpression did not produce significant changes. The glycogenin-like function of CmGLG1 was further verified through a complementation assay using the Saccharomyces cerevisiae CC9 strain, a *glg1glg2* double knockout mutant incapable of glycogen accumulation (Cheng et al., 1995).

**Figure 2:**
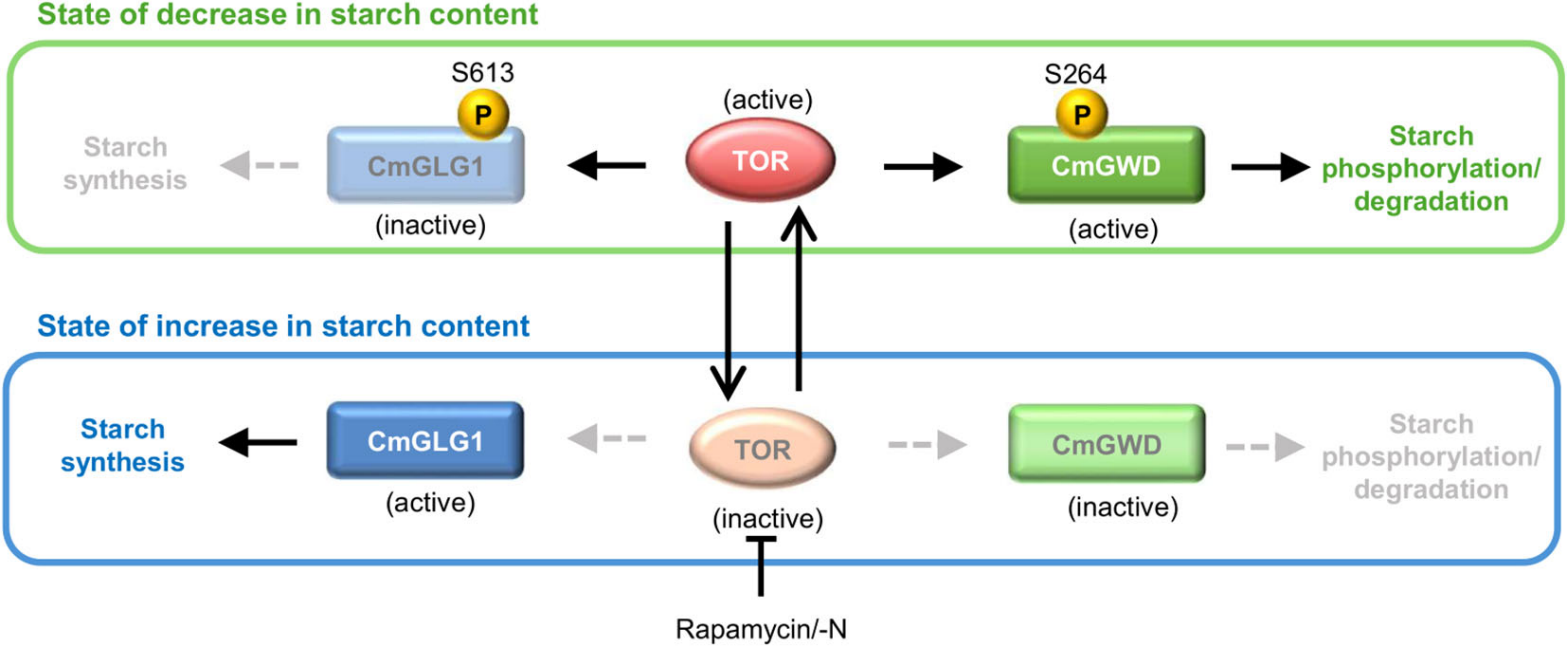
A potential model for the regulation of starch content via TOR signaling in *C. merolae*. This figure illustrates the potential models of starch phosphorylation/degradation and synthesis by TOR signaling. The letter “P” within the yellow circles indicates phosphorylation at specific amino acid residues. The numbers and “S” signify phosphorylation sites and serine, respectively. The closed and open arrowheads represent the effects of the indicated proteins and changes in status, respectively. Blunt arrows indicate inhibition of TOR function/activity. Solid lines represent functional protein activity, whereas grey dashed lines denote reduced or absent protein activity. When the starch content decreases, CmGWD and CmGLG1 are phosphorylated either directly or indirectly through the TOR signaling pathway. This phosphorylation activates CmGWD, allowing it to phosphorylate starch while simultaneously inactivating CmGLG1, which suppresses *de novo* starch synthesis and promotes starch breakdown. In contrast, when TOR signaling is inhibited—such as via rapamycin treatment or nitrogen deficiency (−N)—phosphorylation of CmGWD and CmGLG1 does not occur. Under these conditions, CmGWD becomes inactive, whereas CmGLG1 becomes active. This reduces starch phosphorylation and enhances *de novo* starch synthesis, ultimately resulting in starch accumulation.

LC-MS/MS analysis identified Ser613 as the phosphorylation site in CmGLG1. Phosphorylation at Ser613 decreased by approximately 50% after 24 hours of rapamycin treatment, whereas phosphorylation levels increased under control conditions. To assess the functional impact of Ser613 phosphorylation, strains expressing CmGLG1 variants mimicking phosphorylated and dephosphorylated states were constructed. Starch accumulation assays revealed that the phospho-mimetic variant exhibited a ~60% reduction in starch accumulation compared to wild-type CmGLG1 and the dephospho-mimetic variant. These findings indicate that phosphorylation at Ser613 inhibits starch synthesis, whereas dephosphorylation triggered by TOR inactivation under conditions such as nutrient depletion enhances starch synthesis, leading to starch accumulation (as illustrated in **Figure 2**).

In addition to CmGLG1, *C. merolae* possesses other glycogenin-type proteins, including CMG174C (CmGLG2), CMR358C, and CMJ262C. Yoshida et al. demonstrated that CMR358C and CMJ262C synthesize polyglucans forming division rings in chloroplasts and mitochondria, termed PLASTID DIVIDING RING1 (PDR1) and MITOCHONDRION DIVIDING RING1 (MDR1), respectively (Yoshida et al., 2010; 2017). Although the function of CmGLG2 was initially unclear, its involvement in starch accumulation was elucidated in 2019, when overexpression of CmGLG2 resulted in a two-fold increase in starch content compared to the control strain (Pancha et al., 2019b). Phylogenetic analysis further supports this role, classifying CmGLG2 within a clade associated with glycogen synthesis in yeast and rabbit muscle, whereas CmGLG1 clusters with plant-specific glycogenin-like starch initiation proteins (PGSIPs), whose reduced expression leads to decreased starch levels. PDR1 and MDR1 belong to a distinct clade separate from CmGLG1 and CmGLG2. Collectively, these findings suggest that CmGLG1 and CmGLG2 both contribute to starch synthesis in *C. merolae*. However, whether TOR regulates CmGLG2 in a manner similar to CmGLG1 remains unknown, highlighting an important direction for future research.

#### 4.1.2 Regulation of starch degradation by TOR-signaling

Phosphoproteomic analysis has also provided important insights into the mechanism of starch degradation. In the initial analysis, only carbohydrate metabolism-related proteins that exhibited decreased phosphorylation following TOR inactivation by rapamycin treatment were considered potential TOR substrates (Pancha et al., 2019a). Conversely, proteins whose phosphorylation increased under control conditions but did not increase with rapamycin treatment were excluded from consideration (Pancha et al., 2019a). A subsequent re-analysis of these excluded proteins identified a candidate TOR substrate annotated as “starch-associated protein R1, alpha-glucan water dikinase” (hereafter referred to as CmGWD), corresponding to CMT547C in the *C. merolae* genome database (http://czon.jp). The functionality of CmGWD as an authentic GWD has been demonstrated through reduced starch phosphorylation levels in a CmGWD knockout strain and supported by phylogenetic analyses (Komiya et al., 2025). LC-MS/MS analysis suggested that Ser264 was the most readily phosphorylated site in CmGWD. Therefore, the effect of this phosphorylation site on CmGWD function was investigated. Expression-inducible strains mimicking phosphorylated Ser264 and dephosphorylated Ser264 were generated by substituting Ser264 with aspartic acid and alanine, respectively, using a nitrogen source-dependent induction system (Imamura et al., 2010; 2018). After 24 h of protein expression induction, starch content was quantified and compared with that at 0-hour starch content. The control and wild-type CmGWD-overexpressing strains accumulated approximately 2.5-fold and 2.4-fold more starch, respectively, than at 0 h. In contrast, the phospho-mimetic CmGWD-overexpressing strain showed approximately 0.6 times lower starch accumulation than the wild-type CmGWD-overexpressing strain, whereas the dephospho-mimetic CmGWD-overexpressing strain showed a 3.5-fold increase. Starch phosphorylation (the amount of phosphorylation per starch molecule) was also assessed under the same conditions used for starch measurements. The results indicated that the phospho-mimetic CmGWD-overexpressing strain had significantly higher starch phosphorylation levels (1.5-fold) than the control, whereas the dephospho-mimetic CmGWD-overexpressing strain had significantly lower levels (0.6-fold). These findings indicate that Ser264 phosphorylation in CmGWD reduces starch accumulation, whereas Ser264 dephosphorylation induces starch accumulation by reducing the intracellular starch phosphorylation levels, thereby inhibiting starch degradation (Komiya et al., 2025) (**Figure 2**).

Since TOR and GWD are widely conserved in eukaryotes, the mechanism by which TOR regulates starch accumulation by modulating GWD activity is likely to be broadly conserved among photosynthetic eukaryotes. Although a serine or threonine residue corresponding to Ser264 of CmGWD could not be identified in published red algal GWD sequences, the Ser264 residue is conserved in several crops such as rice, barley, and wheat. This suggests that such regulatory mechanisms are conserved, highlighting an important aspect for improving crop yields, particularly starch production. Improving crop yields, especially starch production, is a significant concern for addressing current global food demands.

### 4.2 Regulation of starch metabolism by overexpression of key genes involved in carbon allocation

Recent work has shown that overexpression of the MYB1 transcription factor in the green alga *Chlamydomonas reinhardtii* markedly enhances the accumulation of both starch and TAG (Zhao et al., 2023). Although MYB1 was originally identified as a factor that induces lipid accumulation under nitrogen-deprived conditions (Choi et al., 2022), Zhao et al. (2023) demonstrated that MYB1 also exerts broad regulatory effects on carbon storage metabolism under standard growth conditions.

In MYB1-overexpressing strains, the expression of multiple genes involved in the starch biosynthetic pathway was upregulated, resulting in a pronounced redirection of carbon flux toward starch synthesis. Specifically, the transcripts of key enzymes directly involved in starch synthesis such as the large and small subunits of AGPase and phosphoglucomutase 1 tended to increase, resulting in a substantial enhancement of intracellular starch accumulation. Such upstream regulation mediated by a transcription factor enables a broad reallocation of metabolic fluxes that is difficult to achieve through modification of single enzymes, highlighting the potential of this strategy for efficiently improving carbon storage capacity in microalgae.

In other recent studies, the co-overexpression of fatty acid transporters (FAX1 and FAX2) and the ABC transporter (ABCA2) influenced lipid metabolism, resulting not only in enhanced cellular lipid content but also in improved starch and biomass accumulation under nitrogen starvation in *C. reinhardtii*. These effects were achieved through the modulation of lipid and starch metabolism and alterations in photosynthetic activity (Chen et al., 2023). Therefore, the overexpression of genes regulating starch content, such as MYB1 and lipid transporters, is regarded as an effective approach to enhance starch accumulation.

However, previous studies in other algae and higher plants suggest that starch accumulation is not necessarily regulated at the transcriptional level (Smith et al., 2004; Pancha et al., 2019a). Therefore, approaches that aim to enhance starch accumulation by overexpressing transcription factors analogous to MYB1 are likely to be highly dependent on the biological characteristics of each organism.

### 4.3 Regulation of starch metabolism by DYRK kinase

Schulz-Raffelt et al. (2016) identified a novel genetic regulator of the accumulation of energy-rich compounds such as starch and oil in the green microalga *Chlamydomonas reinhardtii*. Through a forward genetic screen, the authors identified a mutant strain, *std1*, which exhibited significantly higher levels of both starch and triacylglycerols (TAGs) under nutrient stress, such as nitrogen or sulfur deprivation. Molecular analysis revealed that the mutation disrupted a gene encoding a plant-specific dual-specificity tyrosine phosphorylation-regulated kinase (DYRK), which was termed DYRKP. DYRKP belongs to a previously uncharacterized subgroup within the DYRK family, which is distinct from the well-known DYRK1, DYRK2, and Yak1 kinases. Phylogenetic analysis indicates that this subgroup is unique to plants and algae, suggesting an evolutionarily specialized role in photosynthetic organisms.

In wild-type *Chlamydomonas*, starch and oil accumulation is tightly regulated by nutrient availability and energy status. However, the *std1* mutant bypassed this regulation and accumulated large amounts of starch, even under low light conditions or without acetate supplementation. Another important aspect of this study is that the *std1* mutant not only accumulated more starch, but also maintained higher photosynthetic activity during nitrogen starvation. This indicates that the removal of the repressive function of DYRKP enhances the sink capacity of the cell, allowing photosynthetic energy to be more effectively converted into storage compounds, such as starch, rather than being downregulated under stress. This also results in greater biomass productivity, as shown in the photobioreactor experiments, where the std1 strain accumulated more biomass with a higher amount of starch accumulation, even under nitrogen starvation conditions.

**Figure 3:**
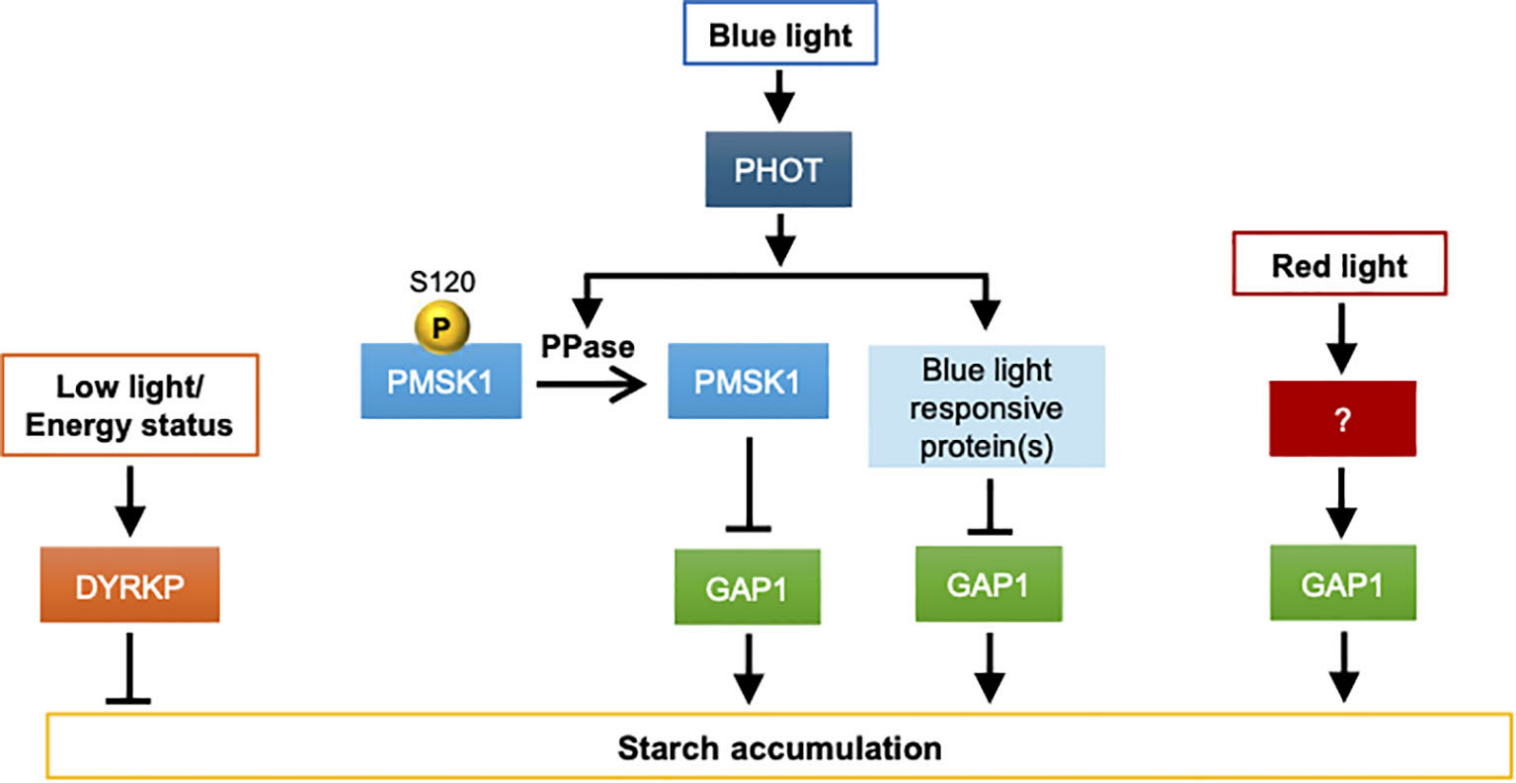
A proposed model for the starch regulation by light quality in *C. reinhardtii*. This figure illustrates the proposed models of starch accumulation and degradation in response to light conditions. The meanings of the lines and other graphical elements shown in the figure are the same as those in **Figure 2**. DYRKP negatively regulates starch accumulation under both nutrient and energy conditions, indicating its critical role in this process. Additionally, light signals play an important role in starch accumulation. Under blue light, phototropin (PHOT) receives the signal and dephosphorylates PMSK1 at serine 120 residue. Dephosphorylated PMSK1 reduces the expression of GAP1 and starch content in cells. PPase is a hypothetical phosphatase that could dephosphorylate phosphorylated PMSK1 in a PHOT-dependent manner. The model also indicates a few phototropin-dependent but PMSK1 independent regulation of starch accumulation under red light conditions, as well as the regulation of starch accumulation through an unknown blue light-responsive protein.

The results of this study indicate that inactivation of DYRKP leads to the decoupling of nutrient signaling from energy reserve accumulation, allowing cells to accumulate higher amounts of starch, even under various stress conditions. This is highly relevant for biofuel production because increasing the starch and oil content in microalgae is a major goal for economically viable algal biofuels. These results indicate that DYRKP is a central regulator of energy storage in *Chlamydomonas*, limiting the accumulation of energy reserves under low-energy conditions (**Figure 3**).

In a recent study by Kim et al. (2025), it was demonstrated that in a double mutant lacking both DYRKP and ADP-glucose pyrophosphorylase (AGP) an essential enzyme involved in starch biosynthesis—the cells exhibited nearly three times higher total fatty acid content compared to the parental strain under nitrogen-replete conditions in *C. reinhardtii*. Furthermore, it has been also reported in microalga *Euglena gracilis* that inhibition of DYRK homologs EgSTD1 and EgSTD2 increase the accumulation on paramylon and wax ester in the cell (Kimura and Ishikawa, 2018). These indicate DYRKP might serve as important control point for carbon partitioning.

### 4.4 Regulation of starch metabolism by phototropin-mediated light signaling

For photosynthetic microalgae, light not only serves as important for photosynthesis but it also important as biological signals for various cellular processes (Yamaoka et al., 2025). Light quality and duration also affect the starch accumulation patterns in microalgae (**Figure 3**). Yuan et al. (2025) reported that the model microalga *Chlamydomonas reinhardtii* accumulates more starch when cultivated under red light than under blue or white light. This indicates that starch accumulation in microalgae may be regulated by photoreceptors. To confirm this hypothesis, they constructed various photoreceptor mutants, namely *acry*, lacking the ANIMAL-TYPE CRYPTOCHROME6, *pcry* lacking the PLANT-TYPE CRYPTOCHROME23, the double *acrypcry* mutant, and the phot mutant devoid of PHOTOTROPIN.

Starch analysis indicated that only the phototropin mutant accumulates almost 3-fold higher starch content than wild-type cells. Further complementation resulted in starch content similar to that of wild-type cells, and removal of the LOV domain of the receptor resulted in less starch accumulation, indicating that phototropin plays an important role in starch accumulation in *Chlamydomonas*.

Further transcriptomic and proteomic analyses indicated that the mRNA levels of chloroplast located GAP1 (glyceraldehyde-3-phosphate dehydrogenase) were almost 80-fold higher in *phot* mutants than in wild-type cells. The overexpression of GAP1 in the *phot* mutant background resulted in higher accumulation of starch, whereas knockdown of GAP1 in the *phot* mutant background resulted in a reduction in starch content, further confirming the important role of GAP1 in starch accumulation through blue light signaling.

To identify the mechanism by which phototropin regulates GAP1 expression, phosphoproteomic analysis was performed. The results showed that the conserved kinase PHOTOTROPIN-MEDIATED SIGNALING KINASE 1 (PMSK1) was highly phosphorylated in the dark and this phosphorylation was rapidly reduced upon blue light illumination in WT cells. However, in the *phot* mutant, PMSK1 remained highly phosphorylated, even after blue light exposure. Serine120 is a key residue that is dephosphorylated in wild-type, but not in *phot* mutants. To confirm this hypothesis, Yuan et al. (2025) constructed FLAG-fused PMSK1. Phos-tag analysis indicated that PMSK1 was rapidly dephosphorylated in the WT but remained phosphorylated in the *phot* mutant. To confirm the role of serine120 residue; phosphomimetic (S120D) and dephosphomimetic (S120A) overexpressing strains were constructed. Overexpression of phosphomimetic strains resulted in higher expression of GAP1 and starch content in both wild-type and *phot* mutants, indicating that the phosphorylation status of S120 in PMSK1 directly controls starch accumulation.

Yuan et al. (2025) constructed the CRISPR-Cas9 based *pmsk1* and *phot pmsk1* double mutants to confirm the role of this kinase. When these mutant lines were grown under different light conditions, the *pmsk1* mutant showed lower GAP1 mRNA and starch content indicates pmsk1 is a positive regulator of starch synthesis. Notably, GAP1 expression in *pmsk1* plants varied with light quality and was higher under red light, suggesting the existence of PMSK1-independent regulators. The *phot pmsk1* double mutant lost this red light–mediated regulation, indicating that PHOT modulates GAP1 expression through additional PMSK1-independent pathways. Another interesting aspect of this study is that high starch accumulation in the phot mutant did not impair growth or photosynthesis under low-light conditions, suggesting that this regulatory pathway modulates carbon allocation without compromising fitness. In case of microalga, *Nannochloropsis oceanica* blue light plays an important role in lipid accumulation through transcription factor NobZIP77 (Zhang et al., 2022; Yamaoka et al., 2025). During sufficient nitrogen conditions, NobZIP77 inhibits the transcription of NoDGAT2B which is type-2 diacylglycerol acyltransferase important for TAG accumulation in microalgae. However, under blue light the binding of NoDGAT2B with transcription factor NobZIP77 is reduced and cells accumulate TAGs (Zhang et al., 2022; Yamaoka et al., 2025). These data indicate light signals have different role in different microalgae.

## 5 Future perspectives

As mentioned in the section of “Mechanisms regulating starch accumulation in microalgae, “ the regulation of starch accumulation in microalgae has been elucidated in certain instances, although a comprehensive understanding of this regulation has yet to be achieved. However, it is evident that TOR plays a critical role in regulating starch accumulation in microalgae. This is because TOR inactivation leads to starch accumulation, a phenomenon observed not only in microalgae, but also in terrestrial plants. In terrestrial plants, recent research by Han et al. (2022) demonstrated that both chemical or genetic inhibition of TOR kinase completely blocks light-induced starch degradation in the guard cells of Arabidopsis leaf epidermal cells. This TOR-dependent inhibition of starch breakdown consequently impairs light-induced stomatal opening. Furthermore, the activation of TOR results in the phosphorylation of Ser31 on BAM1, which stabilizes BAM1 via a downstream kinase (currently unidentified), thereby promoting rapid starch degradation in guard cells. This finding strongly supports the importance and conservation of TOR in the regulation of starch accumulation in plant lineages. Further analyses on how TOR regulates starch accumulation will provide deeper insights into the mechanisms of starch accumulation. Additionally, uncovering the relationship between TOR-dependent regulation and mechanisms involving factors, such as PSR1, DYRKP, and phototropin, will aid in understanding the detailed molecular processes involved in starch regulation. In this endeavor, knowledge gained from studies on both microalgae and land plants will be mutually beneficial for elucidating the regulatory mechanisms of starch accumulation across plant lineages.

Starch accumulates under light and is degraded in the dark, a process influenced by TOR signaling. Recent studies by Mallén-Ponce et al. (2022) demonstrated that in *C. reinhardtii*, TOR activity increases under light conditions and decreases in darkness. Thus, starch accumulation and TOR activity, as well as light/dark conditions and TOR activity, exhibit inverse correlations. Furthermore, although TOR has been shown to influence starch regulation by altering the phosphorylation states of CmGLG1 and CmGWD, it remains unclear whether TOR directly phosphorylates these proteins or acts indirectly through other kinases (**Figure 2**). These findings suggest that TOR-mediated regulation of starch accumulation is complex. Despite numerous studies on starch metabolism in microalgae, our limited understanding of the regulatory networks governing starch synthesis and degradation remains a major limitation. While core enzymes involved in starch metabolism are well characterized, the roles of transcription factors, signaling pathways, and post-translational modifications remain poorly understood in most microalgal species. Achieving a comprehensive understanding will advance the scientific field and promote industrial utilization of algae-derived starch, potentially redirecting more grain-derived starch for food applications and helping address environmental and food-related challenges. Additionally, the dynamic interaction between starch and other carbon storage compounds, particularly lipids, is not fully explored. Understanding how microalgae switch carbon flux between starch and triacylglycerols under various conditions is crucial for designing strains optimized for dual-product biorefineries.
